# Taking rehabilitation seriously

**DOI:** 10.2471/BLT.19.020819

**Published:** 2019-08-01

## Abstract

Under-appreciation of the importance of rehabilitation within the continuum of care is a major obstacle to achieving universal health coverage. Lynn Eaton reports.

Shakya Nishchal remembers the 2015 Nepal earthquake well. “I was at home in Kathmandu with my family when it struck,” he says. “I was thrown against the walls as I ran through to my baby’s room to protect her. It was terrifying.”

Once his family was safe, and the ground had stopped shaking, Nishchal (who is now a senior lecturer at Kathmandu University, but was then chairperson of the Nepal Physiotherapy Association) got in touch with colleagues to organize a rehabilitation team. 

Getting a group of specialists together was not a problem, but getting them into action was. “After the earthquake, despite trying hard at all levels, it was very difficult to set up a service department for physiotherapy, even in the most affected areas,” Nishchal says.

What made it difficult for Nishchal and his colleagues to go to work was not a lack of demand, (an estimated 22 000 people were injured in the earthquake) it was a lack of appreciation on the part of the health ministry of the importance of rehabilitation services in an emergency.

Sunil Pokhrel, a physiotherapist with the nongovernmental agency Humanity & Inclusion, which played a key role in response efforts, encountered similar issues.

“The health ministry had been working on an emergency preparedness plan in collaboration with WHO, so we were not entirely caught off guard, but even so, the clinics and hospitals were quickly overwhelmed with patients,” he says.

According to Pokhrel most of the doctors and nurses had an awareness of rehabilitation services because of training given to them during the preparedness planning. However, there were very few staff trained in rehabilitation and they had limited stocks of assistive devices.

For Pauline Kleinitz, a physiotherapist working with the World Health Organization (WHO), Nepal’s experience highlights challenges faced by many low-income countries.

“Health emergencies such as Nepal’s 2015 earthquake throw into stark relief the problems resource-constrained countries face, notably the tendency to underfund rehabilitation services,” she says.

While there is a lack of robust national studies of unmet rehabilitation need, according to WHO’s *Global atlas of the health workforce,* the density of skilled rehabilitation practitioners, in many low- and lower- middle-income countries is below 10 per 1 million people.

Kleinitz believes there is a link between the under-allocation of resources and the way rehabilitation is defined. “The pigeon-holing of rehabilitation as an ‘extra’ or as something that is only needed for people with significant permanent disability often stands in the way of health system planners making the necessary investment in capacity across the system,” she says.

The exclusive focus on permanent impairment was something that Nishchal observed during the earthquake response. “Those rehabilitation interventions that were implemented, mainly targeted people with permanent impairments, such as lost limbs,” he says.

That a resource-constrained health system under the pressures imposed by an emergency should prioritize the needs of someone who has just lost a leg, first by keeping them alive, and second by helping them adjust to their new reality, is of course understandable. It can also be argued that such a patient, in any circumstances, will always take priority over patients with less pressing needs.

However, it is important to recognize that the benefits of rehabilitation extend well beyond such cases and should be taken into account in capacity building and resource allocation.

Since 2017 Kleinitz has been assisting countries to develop rehabilitation capacity as part of the WHO Rehabilitation 2030 initiative. Launched in 2017, the initiative identifies areas requiring concerted action to reduce unmet needs for rehabilitation and strengthen health systems to provide rehabilitation services. The second Global Rehabilitation 2030 Meeting took place in July 2019, bringing together representatives from 55 countries.

As part of her work Kleinitz has been supporting Botswana, Guyana, Jordan, Myanmar, Nepal, Solomon Islands, and Sri Lanka in their efforts to assess their situation and to develop national plans.

In her advocacy and technical support efforts Kleinitz stresses the importance of ensuring rehabilitation services for a wide range of health conditions. “Rehabilitation addresses much more than disability and is in fact needed by anyone with a health condition, impairment or injury, acute or chronic, that limits functioning,” she says.

Kleinitz argues that for rehabilitation to realize its full potential it needs to be integrated into the broader health system, from the primary health-care (PHC) level up. “People need to be able to access appropriate rehabilitation services in their own town or village so that they don’t always have to travel to a specialist centre based in a major hospital,” she says, and argues that integrating rehabilitation into PHC is key to advancing the universal health coverage agenda, and progress towards the broader health goal (Sustainable Development Goal 3).

Kleinitz stresses however, that optimal delivery of rehabilitation services requires them being integrated into and between primary, secondary and tertiary levels of health systems, as well as with social welfare services and long-term care.

Needless to say, the integration of rehabilitation services across health systems is predicated on the existence of health systems that can deliver a full range of health services and have effective referral systems.

Most of the low- and middle-income countries Kleinitz is working with fall short on both counts.

Myanmar is one example. According to a recent WHO assessment of the country, over the past decade, health spending has increased and there has been a corresponding expansion of government health services. However, legacy weaknesses accumulated during several decades of military rule are still very evident.

Despite the challenges faced, over the last decade rehabilitation capacity has improved. There are now 23 physical medicine and rehabilitation departments across the country’s 38 tertiary hospitals, while 76% of the secondary level hospitals have at least physiotherapy services.

“Rehabilitation is being increasingly integrated into a range of tertiary and secondary healthcare, but there is still progress to be made,” says Kleinitz, citing the limited integration of rehabilitation within primary healthcare as an example. “Very few rehabilitation personnel are located at the primary care level, nor have rehabilitation interventions been built into the provision of primary health care by other health personnel,” she says.

“Rehabilitation… needs to be integrated into the broader health system, from the primary health-care level up.”

Pauline Kleinitz

Dr Myint Htwe, Myanmar’s health minister, acknowledges the need for increased investment in rehabilitation services, and considers WHO’s assessment of the current state of rehabilitation service provision ‘a wake-up call’.

Myint reports that the government plans to train health workers in primary health care centres to provide basic rehabilitation advice, but acknowledges that this may take time. In the short term there are plans to boost capacity by making smart phones available to staff that will support diagnosis and treatment. The government has already circulated 12 000 smart phones and plans to distribute another 14 000 in August.

President of the Myanmar Physiotherapy Association, Tin Hlaing Soe believes that bringing in private sector players could also help. “The government should train more physiotherapists and create specialized posts in small towns as well as encouraging private clinics,” he says.

In Nepal the resource constraints are just as daunting. Despite various initiatives to support health systems strengthening, dating back to the promulgation of a national health policy in 1991, and despite Nepal’s 2015 constitution guaranteeing basic health care as a fundamental right, to date the development of a robust health system has been more an aspiration than a reality.

With specific regard to rehabilitation, it is grass roots efforts that have driven the rehabilitation agenda, notably the community-based rehabilitation schemes, which are now established in each of the country’s 75 districts.

“Under the direct control of the local community and disabled persons’ organizations, these schemes have played an important role in promoting inclusion and in collecting basic information related to persons with disabilities,” Kleinitz says, noting that many are now moving beyond rehabilitation to include services and initiatives designed to promote inclusion of persons with disabilities.

However, while Kleinitz applauds these initiatives, she is keen to see the governments build on the health system capacity developed in response to the earthquake.

Nishchal shares the same desire. “The seven health facilities equipped with rehabilitation services after the quake still continue to provide rehabilitation care to people with injuries and limitations in functioning caused by back pain and osteoarthritis,” he says, adding that they are still actively involved in early detection, assessment and treatment, follow-up through community home visits and outreach programmes. “This has set an example in the country,” he says. “They offer a ray of hope.”

**Figure:**
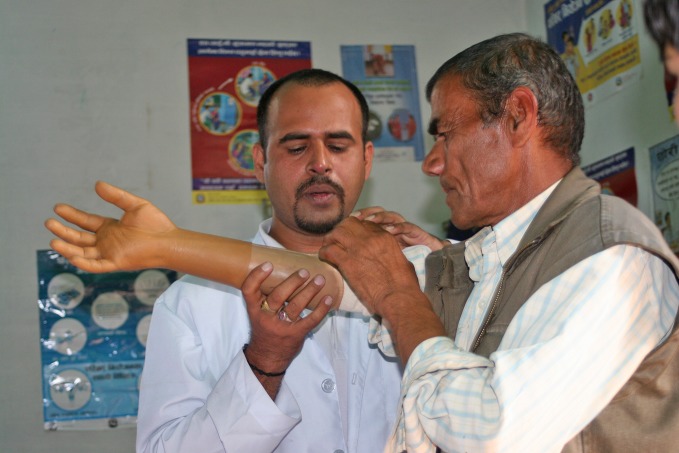
A Nepalese patient is fitted with a prosthesis.

WHO/Sue Eitel

**Figure:**
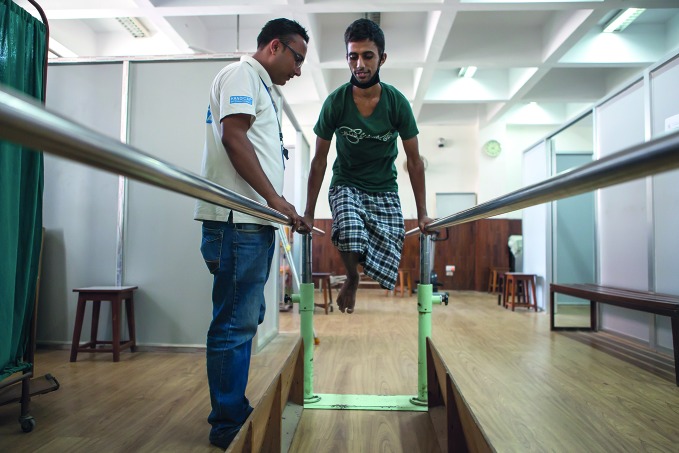
A Nepalese patient working with physiotherapist Sunil Pokhrel.

Humanity&Inclusion/Lucas Veuve

